# Bilateral subdural hematoma caused by spontaneous intracranial hypotension originating from a discogenic microspur successfully treated with duraplasty: A case report

**DOI:** 10.1016/j.bas.2022.100879

**Published:** 2022-03-11

**Authors:** Andrea Casanova, László Entz, Simon Weinmann, Isabel Wanke, Robert Reisch

**Affiliations:** a University of Zurich, Rämistrasse 71, 8006, Zurich, Switzerland; b Department of Neurosurgery, Hirslanden Klinik, Witellikerstrasse 40, 8032, Zurich, Switzerland; c Department of Emergency Medicine, Hirslanden Klinik, Witellikerstrasse 40, 8032, Zurich, Switzerland; d Department of Radiology, Hirslanden Klinik, Witellikerstrasse 40, 8032, Zurich, Switzerland

**Keywords:** Discogenic microspur, Cerebrospinal fluid leak, Spontaneous intracranial hypotension, Subdural hematoma, Duraplasty

## Abstract

**Introduction:**

Discogenic microspurs are calcified outgrowths from the intervertebral disc which can perforate the dura, causing a leak of cerebrospinal fluid (CSF). Spontaneous leaks of the CSF present a recognized cause of spontaneous intracranial hypotension (SIH). Moreover, subdural hematomas (SDH) are a potentially severe complication of SIH.

**Research question:**

We present a case of a bilateral subdural hematoma without orthostatic headaches caused by a discogenic microspur protruding from the T1-2 intervertebral disc. The microspur is conjectured to be the culprit of the leak by ventrally perforating the dura and catalyzing the causal chain leading to the formation of the subdural hemorrhage.

**Material and methods:**

A 79-year woman noticed a progressive gait disturbance accompanied by a decline of short-term memory over several months without experiencing orthostatic headaches. Magnetic resonance imaging (MRI) showed extensive bilateral subdural fronto-parietal hematoma, signs of CSF hypotension (dilated venous compartments), and computed tomography (CT) myelography revealed a CSF leak originating at the T1-2 level.

**Results:**

The leakage site was treated with microsurgical duraplasty leading to a regression of the symptoms and complete resolution of the subdural hematomas within five postoperative months.

**Discussion and conclusion:**

Discogenic microspurs can perforate the dura causing a CSF leak, leading to spontaneous intracranial hypotension, finally resulting in a bilateral subdural hematoma. This constellation of symptoms does not necessarily induce orthostatic headaches and can be treated with microsurgical duraplasty.

## Highlights

• Discogenic microspur perforating the dura and inducing a liquor leak.
• Discogenic microspur leading to spontaneous intracranial hypotension.
• Discogenic microspur leading to bilateral subdural hematoma.
• Liquor leak treated by microsurgical duraplasty.
• Bilateral subdural hematoma retraction after microsurgical duraplasty.

## 1 Introduction

Hypovolemia of the cerebrospinal fluid (CSF) can result in various clinical manifestations. These can span a wide range from entirely asymptomatic to orthostatic headaches to neurological abnormalities up to death (Ferrante et al., 2018; Chung et al., 2000; Mokri, 1999). A well-known occurrence is a post-dural-puncture headache following accidental iatrogenic dural perforation in peridural anesthesia (Ghaleb, 2010). A less common phenomenon is spontaneous intracranial hypotension (SIH) which, due to its relatively unspecific clinical appearance, unclear trigger, and slow onset, remains a challenge to diagnose. Untreated SIH can lead to complications like subdural fluid collection or potentially life-threatening subdural hematomas (SDH) (J and L, 2018).

Spinal fluid leakage is increasingly recognized to contribute to SIH (Schievink, 2006; Beck et al., 2016). Whenever a loss of CSF is suspected, it is crucial to identify its cause to initiate appropriate therapeutic approaches. (Schievink et al., 2016). CSF leaks due to SIH are typically solitary, with multiple leaks recorded either at the (postero)- lateral location of the dura or with CSF-venous fistulas (Schievink et al., 2021).

Recently established MRI-based scores may aid in quantifying the likelihood of having a CSF leak due to SIH, warranting further myelography (Dobrocky et al., 2019, 2022). However, no MRI finding is pathognomonic (Dobrocky et al., 2019). When detected early and treated Dobrocky et al., 2019, 2022accordingly, SIH may resolve by conservative methods (bed rest, oral hydration, caffeine, steroids). Nevertheless, in some cases, invasive procedures like repeated epidural blood patching (EBP) or duraplasty are required to achieve relief (Ferrante et al., 2018; Schievink, 2006; Yoshida et al., 2014; Chen et al., 2008).

A discogenic microspur (DM) is a calcified outgrowth of the intervertebral disc, which can surface as calcification of the disc progresses, accompanied by a reaction to increased mechanical stress (Beck et al., 2016; Rogers et al., 1997). Protrusions of the intervertebral disc represent a recognized cause of dural tear (Beck et al., 2016). Among spinal osteophytes (Thielen et al., 2015; Hung and Hsu, 2015) and herniated discs (Rapport et al., 2003; Allmendinger and Lee, 2013), a DM is the main perpetrator of ventral CSF loss by creating vertical longitudinal dural slits. Due to its small size and typically ventral location, its presence may easily escape standard diagnostics. Moreover, it can be present at more than one location on the vertebral column (Beck et al., 2016; Thielen et al., 2015).

In most cases with dural tear, a DM is found to be involved (71% (Beck et al., 2016), 67% (Yoshida et al., 2014)), and notably, all ventrally located CSF leaks were determined to be caused by a DM (Beck et al., 2016). Other locations of dural tear were discovered to be due to longitudinal slits on the lateral aspect of the dura in the region of the nerve root axilla (22% (Beck et al., 2016)), typically due to meningeal diverticula, next to a dorsal dural slit caused by a spinal osteophyte (7% (Beck et al., 2016)). While a DM may present at several locations, multiple CSF leaks due to SIH were never recorded in ventral leaks and found to be uncommon at other locations (Schievink et al., 2021).

We present a case where the following events act as a causal chain: A DM ventrally produces a dural tear, which induces a CSF leak at the T1-2 level, leading to SIH, and finally resulting in a chronic bilateral subdural hematoma without orthostatic headaches.

## 2 Case report

A 79-year-old woman noticed a progressive gait disturbance accompanied by a short-term memory decline over several months, along with a mild tension in the head without a history of trauma. Her gait followed a broad-based pattern, accompanied by a neurogenic bladder dysfunction. Her performance on the Montreal Cognitive Assessment indicated a mild cognitive impairment and corroborated a deficit of short-term memory. She experienced no orthostatic headaches, no focal deficits, and showed no pyramidal-tract signs. As the patient had been suffering from secondary progressive multiple sclerosis (MS) for over twenty years, with the last episode over twenty years ago, brain magnetic resonance imaging (MRI) was used to search for changes stemming from an acute episode of MS or some other neurodegenerative process.

**Fig. 1:**
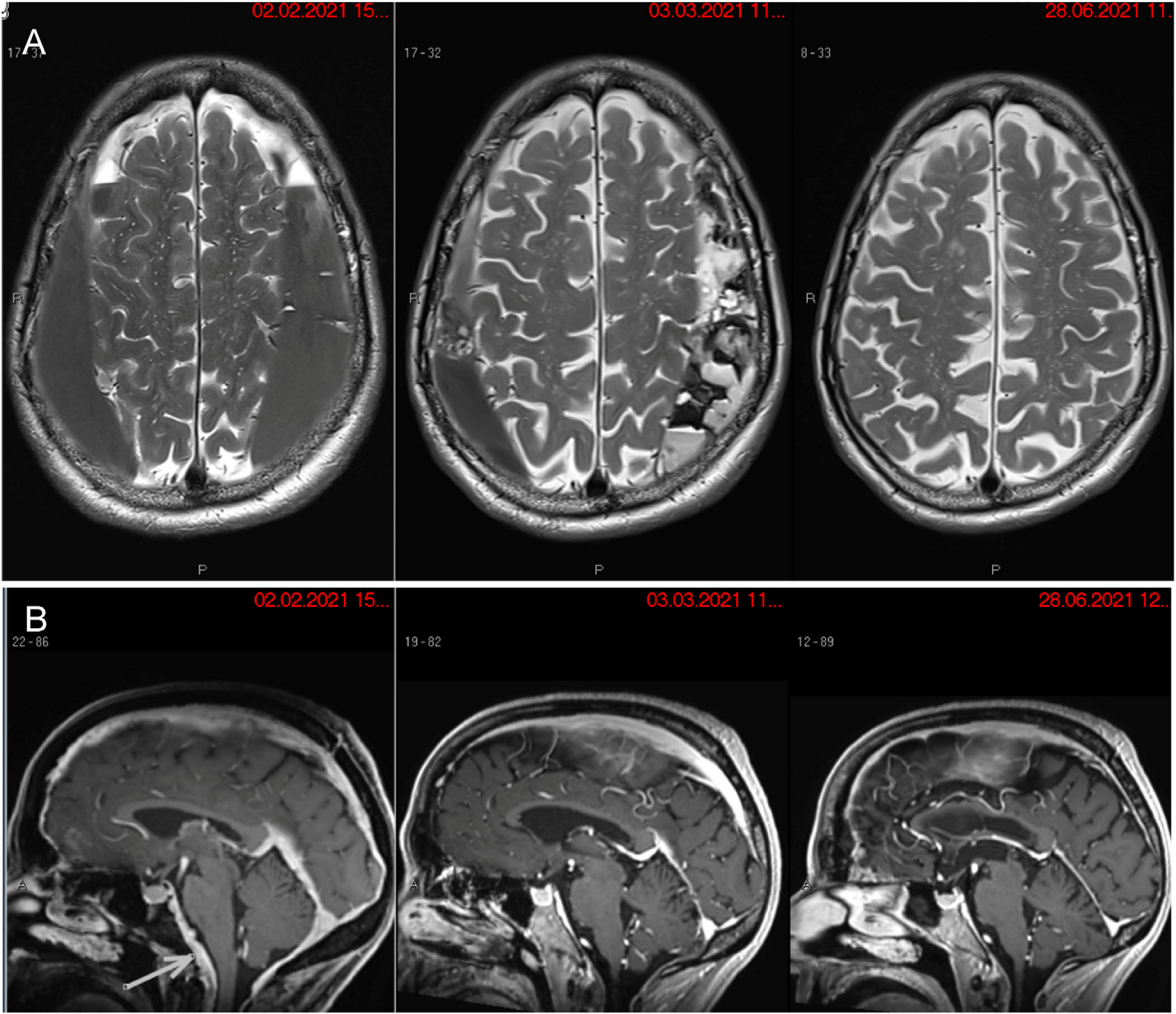
(**A**) Axial MRI images of the brain displaying the course of regression of a bilateral subdural hematoma in the fronto-parietal region, leading to a compression of both cerebral hemispheres without a midline shift. (Left) Six days preoperative. (Middle) Three weeks postoperative, a slight subsidence of fluid accumulation was observed with a normalization of the ventricular system and reappearance of fronto-parietal cerebral sulci. (Right) Five months postoperative with complete regression of the large subdural hematomas. (**B**) Sagittal MRI images of the brain showing regression of venous compartment enlargement due to a liquor-leak syndrome. (Left) Four days preoperative. (Middle) Three weeks postoperative. (Right) Five months postoperative.

Surprisingly, a brain MRI revealed a bilateral subdural hematoma in the fronto-parietal region with sizes ranging from 18 mm width on the left side and 13 mm width on the right side, leading to a compression of both cerebral hemispheres and a reactive supra- and infratentorial dural thickening without a midline shift (Fig. 1). Further findings consisted of known periventricular, supra- and infratentorial MS lesions. The patient was diagnosed with a chronic bilateral SDH due to SIH.

**Fig. 2:**
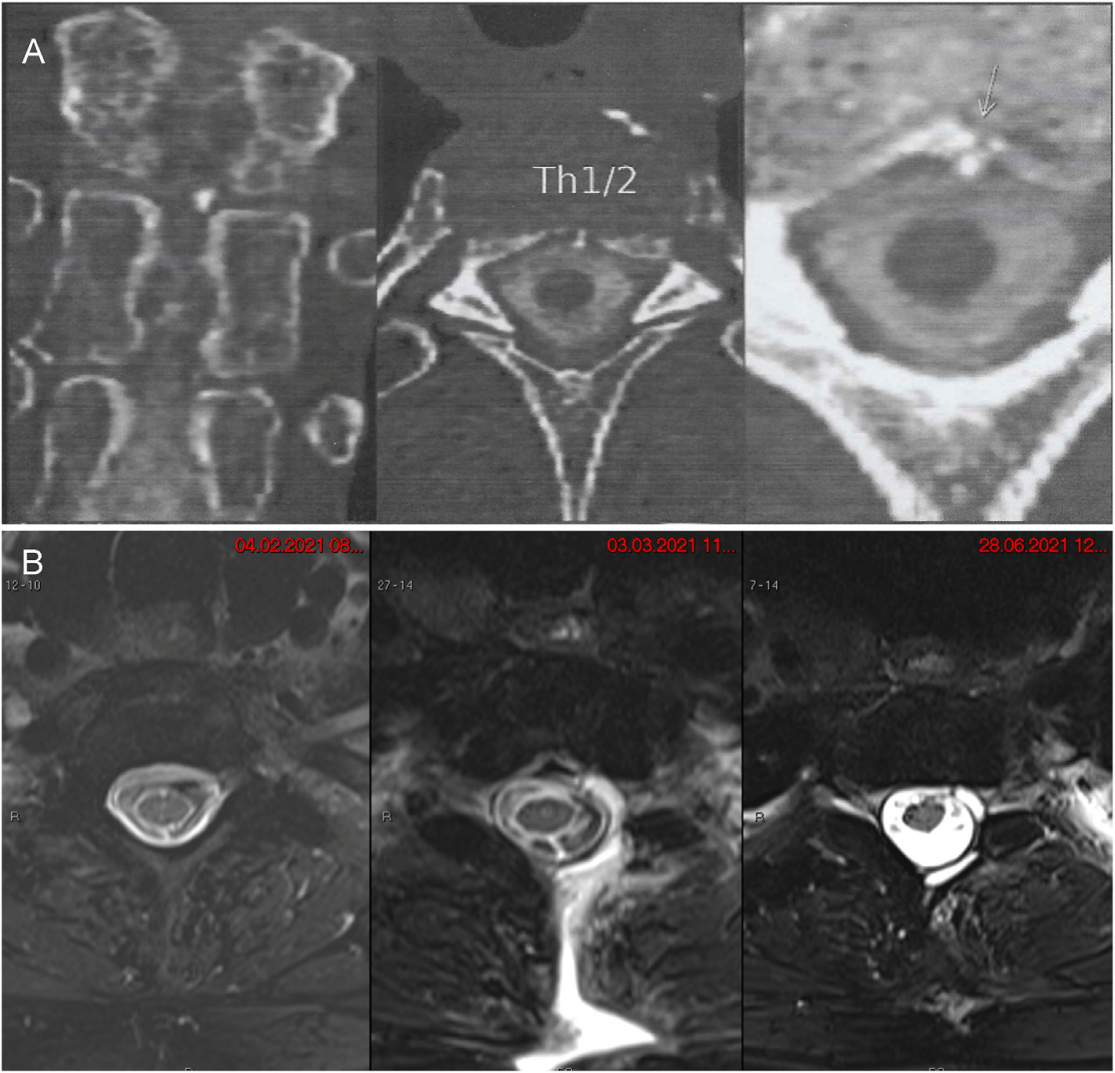
(**A**) CT myelography of the cervicothoracic spine four days preoperative, with a coronal and axial view of a DM out of the intervertebral disc at the T1-2 level, which was assumed to be responsible for the dural leak. (**B**) MRI of the cervicothoracic spine. (Left) Four days preoperative. (Middle) Three weeks postoperative with fluid extravasation at the T1-2 level. (Right) Five months postoperative with complete regression of the epidural leakage.

To search for the cause of the SIH, the patient underwent an MRI of the entire spinal column, which exposed a circular CSF extravasation around the dural sac from the C3- to the T10 level (Fig. 2). In a subsequent computed tomography (CT) myelography of the thoracic spine, a leakage site at the T1-2 level was localized, and a DM on the intervertebral disc at the same level was discovered. Additionally, to the T1-2 level, where a calcified microspur was discovered, the intervertebral disc at the T2-3 level showed localized calcifications (Fig. 2).

**Fig. 3:**
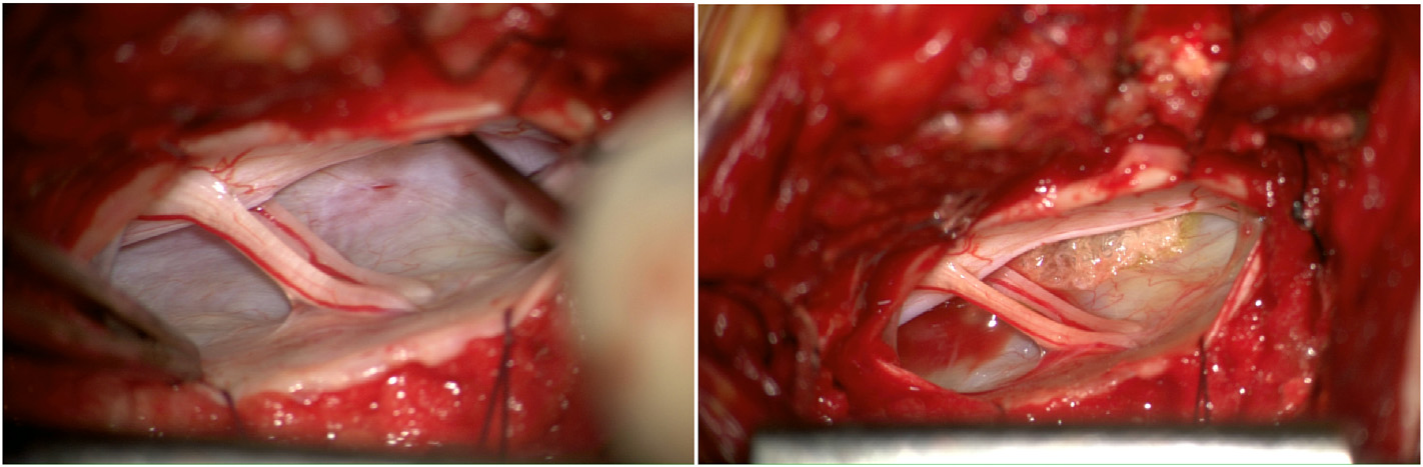
Intraoperative endoscopic view of the tear accessed through an interarcuate fenestration at the T1-2 level (left) and its coating with TachoSil(©), a fibrinogen-thrombin sealant patch (right).

A DM-induced dural tear revealed a persistent mechanical problem. Neither conservative treatment nor an EBP would have promised relief. Therefore, it was decided to perform an endoscopically assisted microsurgical closure of the ventral dural dehiscence by duraplasty accessed through an interarcuate fenestration at the T1-2 level (Fig. 3).

A CT of the skull on the second postoperative day revealed no change in the volume of the SDH. Subdural bifrontal and interhemispheric air inclusions were spotted, most likely due to a postoperative reallocation of air. Five days later, the MRI of the skull showed a reduction of the SDH by 4 mm on the left side and 2–3 mm on the right side with a normalization of the ventricular system and reappearance of frontoparietal cerebral sulci (Fig. 1).

The patient reported a general improvement and experienced less tension in the head, despite the SDH not fully receding during the hospital stay. The patient was discharged on the 13th day of hospitalization without new neurological deficits. Four weeks after surgery, the CSF extravasation subsided slightly, and there was no recurrence detected on brain MRI (Fig. 1). In the next five postoperative months, we witnessed a complete regression of the SDH accompanied by a total normalization of symptoms, including a subjective improvement of short-term memory.

## 3 Discussion

The etiology of CSF leaks often remained undetermined or was prone to initial misdiagnosis in the past, delaying appropriate therapy (Chen et al., 2008). Increasingly, light is being shed on their etiology. A recent study (Schievink et al., 2016) categorized the origins of CSF leaks into 1) dural tear due to an extradural pathology (DM, spinal osteophyte), 2) meningeal diverticula due to a dural pathology, 3) rare cases of CSF-venous fistulas, and 4) undetermined reasons.

Several risk factors can contribute to dural tear. For instance, a weak area around the dura mater, spinal trauma, and connective tissue disorders (Schievink, 2006; Beck et al., 2016). Moreover, Marfan syndrome, Ehlers-Danlos syndrome type II, and autosomal dominant polycystic kidney disease all predispose to SIH (Schievink, 2006; Beck et al., 2016; Chen et al., 2008).

Dural lesions due to microspurs or osteophytes were found widely between C5 and L1, with most lesions at the cervicothoracic level (Hung and Hsu, 2015). In a screening of patients suffering CSF leaks due to microspurs, many patients were found to have multiple calcified microspurs while having only one leak-site (Thielen et al., 2015). SIH was determined to be therapy-resistant in 10–30% of cases despite repeated EBP (J and L, 2018). In another study (Beck et al., 2016), SIH was found to be intractable in 20% of cases. All intractable cases exhibited CSF leaks and 71% were found to have microspurs. Similarly (Yoshida et al., 2014), determined that ventral lesions due to DM caused CSF leaks in 67% of cases. Those patients who underwent conservative methods and repeated EBP required a surgical repair after identifying the exact site of CSF leakage by neuroimaging (typically CT myelography) (J and L, 2018).

The various etiologies of CSF leaks complicate diagnostics and choice of intervention. In our case, the first MRI of the skull only exposed the bilateral SDH, while its underlying cause remained hidden. Based on this finding alone, the neurosurgical guidelines would have mandated evacuating the SDH, only to have found it resurge down the road due to the persisting dural leak. Moreover, postponing an intervention might have introduced further procedures down the line. The cause and location of the CSF leak were revealed in spinal imaging, and the decision for surgical intervention with duraplasty was made.

SIH has an annual incidence of 5 per 100,000 of SIH (Schievink, 2006; Chen et al., 2008). With the high prevalence of DM in intractable cases, the search for microspurs is recommended. They appear to be the predominant cause in refractory SIH cases not responding to conservative therapy or repeated EBP (Ferrante et al., 2018; Beck et al., 2016). In those cases, an intervention with microsurgical methods is required (Beck et al., 2016).

Whenever SIH occurs, it may result in SDH. The Monroe-Kellie doctrine states that the voluminal sum of intracranial blood, CSF, and cerebral tissue remains constant within an intact cranium (Fishman and Dillon, 1993). Hence, every loss of CSF from the spine must either be compensated by increasing the vascular component, resulting in pachymeningeal enhancement, enlargement of venous structures (Schievink, 2006), or by increasing the intracranial CSF volume, leading to the accumulation of CSF in subdural hygromas.

Whenever the loss of CSF from the spine outweighs the compensatory capacity of the vascular components, hematomas may ensue. As the intracranial pressure decreases due to an efflux of CSF, the loss of CSF buoyancy can lead to a downward displacement of the brain, resulting in a rupture of bridging veins pulled away from the dura (Markwalder, 1981). The same outcome can be observed when a gradual increase of the subdural space by a collection of CSF predisposes to tearing of bridging veins or to rupturing the dilated thin-walled blood vessels (Schievink, 2006). Both ways of pathogenesis can result in subdural hematoma, which can be widespread in some cases, as seen in our patient experiencing a bilateral subdural hematoma.

A small study found that in two-thirds of patients, a CSF leak led to a SDH, which was bilateral in 62.5% of cases (Yoshida et al., 2014). Another study found that one-fourth of SDH patients experienced prior subdural hygroma (Lai et al., 2007). Furthermore, the interval of SDH formation caused by a CSF leak ranged from 20 to 99 days (Lai et al., 2007).

Although orthostatic headaches appear as a clinical hallmark for SIH diagnosis, nearly half of patients suffering from SIH have atypical headaches (Mea et al., 2009). Surprisingly, our patient did not suffer from orthostatic headaches, merely noticing an occasional sensation of increased tension in the head. Lastly, the gait disorder of our patient can be explained by the disturbed dynamic equilibrium induced by increased pressure on the cortex by the hematoma, as seen in normal pressure hydrocephalus when enlarged ventricles lessen the cortex distance between cortex and skull.

## 4 Conclusion

We describe a case with spontaneous intracranial hypotension due to a discogenic microspur at the thoracic spine perforating the dura, resulting in chronic bilateral subdural hematoma without orthostatic headaches, which was successfully treated with microsurgical closure by duraplasty.

## Funding

The authors received no financial support for the research, authorship, and/or publication of this article.

## Conflict of interest

The authors have no conflict of interest to declare.
